# Timing of vasopressor initiation and mortality in septic shock: a cohort study

**DOI:** 10.1186/cc13868

**Published:** 2014-05-12

**Authors:** Vance Beck, Dan Chateau, Gregory L Bryson, Amarnath Pisipati, Sergio Zanotti, Joseph E Parrillo, Anand Kumar

**Affiliations:** 1 Department of Anesthesiology, Ottawa Hospital/University of Ottawa, 501 Smyth Road, Ottawa, ON K1H-8L6, Canada; 2 Department of Community Health Sciences, University of Manitoba, S113 Medical Services Bldg, 750 Bannatyne Ave, Manitoba, MB R3E-0W3, Canada; 3 Department of Medical Microbiology, University of Manitoba, Room 543 Basic Medical Sciences Bldg, 745 Bannatyne Ave, Manitoba, MB R3E-0J9, Canada; 4 Section of Critical Care Medicine, Cooper University Hospital, Rowan School of Medicine, 1 Cooper Place, Camden, NJ 08103, USA; 5 Department of Medicine, Hackensack Medical Center, Rutgers New Jersey Medical School, 30 Prospect St., Newark, NJ 07601, USA; 6 Section of Critical Care Medicine, JJ-399, Health Sciences Centre, University of Manitoba, 700 William Ave, Manitoba, MB R3A-1R9, Canada

## Abstract

**Introduction:**

Despite recent advances in the management of septic shock, mortality remains unacceptably high. Earlier initiation of key therapies including appropriate antimicrobials and fluid resuscitation appears to reduce the mortality in this condition. This study examined whether early initiation of vasopressor therapy is associated with improved survival in fluid therapy-refractory septic shock.

**Methods:**

Utilizing a well-established database, relevant information including duration of time to vasopressor administration following the initial documentation of recurrent/persistent hypotension associated with septic shock was assessed in 8,670 adult patients from 28 ICUs in Canada, the United States of America, and Saudi Arabia. The primary endpoint was survival to hospital discharge. Secondary endpoints were length of ICU and hospital stay as well as duration of ventilator support and vasopressor dependence. Analysis involved multivariate linear and logistic regression analysis.

**Results:**

In total, 8,640 patients met the definition of septic shock with time of vasopressor/inotropic initiation documented. Of these, 6,514 were suitable for analysis. The overall unadjusted hospital mortality rate was 53%. Independent mortality correlates included liver failure (odds ratio (OR) 3.46, 95% confidence interval (CI), 2.67 to 4.48), metastatic cancer (OR 1.63, CI, 1.32 to 2.01), AIDS (OR 1.91, CI, 1.29 to 2.49), hematologic malignancy (OR 1.88, CI, 1.46 to 2.41), neutropenia (OR 1.78, CI, 1.27 to 2.49) and chronic hypertension (OR 0.62 CI, 0.52 to 0.73). Delay of initiation of appropriate antimicrobial therapy (OR 1.07/hr, CI, 1.06 to 1.08), age (OR 1.03/yr, CI, 1.02 to 1.03), and Acute Physiology and Chronic Health Evaluation (APACHE) II Score (OR 1.11/point, CI, 1.10 to 1.12) were also found to be significant independent correlates of mortality. After adjustment, only a weak correlation between vasopressor delay and hospital mortality was found (adjusted OR 1.02/hr, 95% CI 1.01 to 1.03, *P* <0.001). This weak effect was entirely driven by the group of patients with the longest delays (>14.1 hours). There was no significant relationship of vasopressor initiation delay to duration of vasopressor therapy (*P* = 0.313) and only a trend to longer duration of ventilator support (*P* = 0.055) among survivors.

**Conclusion:**

Marked delays in initiation of vasopressor/inotropic therapy are associated with a small increase in mortality risk in patients with septic shock.

## Introduction

Despite advancements in understanding and treatment, septic shock remains a worldwide healthcare problem. With an increasing annual incidence in the developed world, mortality remains between 25 and 50% of those afflicted [1-3]. The pathophysiology of septic shock is complex and involves vasodilatation, relative and absolute hypovolemia, myocardial dysfunction, increased metabolic rate and altered regional and microvascular blood flow [4-11]. Septic shock appears to cause a loss of autoregulation, making the perfusion of many vital organs and tissues dependent on blood pressure [5,12,13]. Early and aggressive fluid resuscitation of sepsis has been suggested to have a critical role in optimization of organ perfusion, preservation of end organ function and improvement of survival [14].

Hypotension despite adequate fluid resuscitation therapy is a defining criterion in the diagnosis of septic shock [15]. To maintain organ perfusion, current guidelines recommend maintaining a mean arterial pressure (MAP) of 65 mmHg with fluid therapy and vasopressors even when hypovolemia has not yet been resolved [15]. According to the Surviving Sepsis Campaign this recommendation is considered ‘strong’ although supporting evidence is considered ‘weak’ [15].

Many studies have compared different vasopressor agents for the resuscitation of septic shock but very few have investigated the role that the timing of vasopressor initiation in relation to hypotension onset plays in outcome [16,17].

## Methods

### Study design

Data from a retrospective review of adult patients (≥18 years old) diagnosed with septic shock was used to create the Cooperative Antimicrobial Therapy of Septic Shock Database (member listing in Additional file 1). Consecutive adult septic shock patients from 28 medical institutions in Canada, the United States and Saudi Arabia for periods between 1996 and 2008 were retrospectively identified using either internal ICU registries/databases and/or International Classification of Diseases (ICD-9 or ICD-10) coding strategies. Patients from surgical, medical and mixed ICUs were included. Each potential case was screened to determine eligibility to meet the criteria for septic shock as described by the 1991 Society of Critical Care Medicine/American College of Chest Physicians consensus statement on sepsis definition [18]. All included cases were required to have no other obvious cause of shock. Each institution contributed a minimum of 50 cases. A waived consent protocol was approved by the Health Ethics Board of the University of Manitoba and at each individual participating center (listing in Additional file 2). The Ethics Boards waived the need for informed consent because of the retrospective, risk-free nature of the study in combination with the use of de-identified data.

### Data management

Data including the time to vasopressor administration after documentation of persistent or recurrent hypotension refractory to fluid administration were retrospectively collected from clinical records using a uniform data extraction template by several trained research nurses or research assistants with medical training (medical students, residents, fellows). All data extractors reviewed >100 charts.

Hypotension was defined as a mean blood pressure <65 mmHg, a systolic blood pressure <90 mmHg, or a decrease in systolic pressure of 40 mmHg from the patient’s baseline consistent with the Society of Critical Care Medicine/American College of Chest Physicians criteria for septic shock [18]. An episode of hypotension was considered to represent the initial onset of septic shock when hypotension persisted from the onset despite fluid (>2 l saline or equivalent) administration (persistent hypotension), or when hypotension was only transiently improved (hypotension resolution for <1 hour) with fluid resuscitation (recurrent hypotension). Hypotension that resolved following fluid resuscitation alone (crystalloid or colloid) without subsequent clinical deterioration was not considered to represent the initial onset of septic shock-related hypotension. Similarly, patients exclusively treated with an inotropic agent without a vasopressor during the first 24 hours were excluded from the database. Organ failure was determined according to previously described criteria [3,19].

### Statistical analysis

Statistical analysis was performed using SAS version 9.1 (Cary, NC USA). Descriptive statistics were used to characterize the patient population, including mean and standard deviation for continuous variables (or median and inter-quartile range for skewed distributions) and frequency and proportion for categorical variables. Empirical logit plots were used to explore the functional form of the association between vasopressor delay fraction (analyzed continuously and also as categorized at decile cutpoints) and survival to hospital discharge. The shortest time delay decile (≤6 minutes) was excluded from the analysis as this usually represents cases where hypotension existed for an unknown period before arrival in the emergency department. In this circumstance, the true time from hypotension onset to vasopressor initiation is indeterminate.

The unadjusted association between survival to hospital discharge and vasopressor delay was estimated using simple logistic regression. A similar analysis was done with respect to the occurrence of individual and total number of organ failures after the day of shock (incremental organ failures from day 2 to day 10). A wide variety of epidemiologic factors (age, sex), comorbidities (AIDS, hematologic malignancy (lymphoma/leukemia/multiple myeloma), metastatic cancer, heart disease, organ transplant, hypertension, respiratory disease, renal disease, diabetes, autoimmune conditions, thromboembolism, neurological diseases), severity of illness (Acute Physiology and Chronic Health Evaluation (APACHE) score) [20], laboratory values (admission lactic acid and bicarbonate levels, white cell count) and therapeutic elements (time to initial appropriate antimicrobial therapy) were first assessed with respect to hospital survival and organ failure using univariate analysis. Those that were significant at *P* < 0.05 were retained for inclusion in the model. Multivariable logistic regression was then used to estimate the adjusted association and to identify independent correlates of mortality and organ failure. Mortality and individual organ failure results are expressed as odds ratios (ORs) with 95% confidence intervals (CIs). Total incremental organ failure after the admission day (day 2 to day 10) was analyzed using Poisson regression with results expressed as rate ratios. Because hospital length of stay (LOS) and ICU LOS are count variables, these secondary outcomes were analyzed using generalized linear regression with a negative binomial distribution and logarithmic link function, adjusted for the same covariates as in the primary outcome analysis. Data are expressed as mean ± standard deviation or median with interquartile range as appropriate.

## Results

There were a total of 8,670 patients that fit the diagnostic criteria for septic shock. Thirty patients did not have a time of vasopressor initiation available and were excluded. Another 2,126 patients were excluded due to inadequate data acquisition of other significant analytic variables, primarily time to appropriate antimicrobial therapy from documentation of hypotension. In total, 6,514 observations were included in this analysis.

### Demographic characteristics and existing comorbidity

The baseline characteristics of the patients in the entire cohort are presented in Table 1. The average age was 62 ± 1 years with male predominance (57.0%). The most common existing comorbidities were diabetes inclusive of oral hypoglycemic and insulin-requiring (26.6%), chronic renal failure inclusive of dialysis (23.6%), and hypertension (19.1%). Illness severity is presented in Table 2 with the average APACHE II score being 26.1 ± 8.2. Baseline (day 1) laboratory results also presented in Table 2 showed elevated levels of serum creatinine (219 ± 181 μmol/l), leukocyte count (16.3 ± 16.1 × 10^6^ cells/l), International Normalized Ratio (1.5 ± 1.4) and serum lactate (4.8 ± 4.4 mmol/l). The heart rate was elevated at 115 ± 29 beats/minute. Approximately 40% of cases were due to nosocomially acquired infection (Table 2). Culture negative and bacteremic/fungemic patients each accounted for about one-third of the cohort. The lungs, abdomen and urinary tract were the most common infection sites and *Escherichia coli*, *Staphylococcus aureus* and *Streptococcus pneumoniae* were the most frequently isolated pathogens (Table 2).

**Table 1:** **Epidemiologic characteristics of the study cohort (*n*** **= 6,514)**

**Characteristic**	**Number**	**Percentage**
Male gender	3,711	57.0
Age (years)^a^	62.1 ± 16.1	
Comorbid disease		
AIDS	176	2.7
Lymphoma	238	3.7
Leukemia	347	5.3
Metastatic cancer	566	8.7
Immunosuppressed	959	14.7
Neutropenia	321	4.9
Liver failure	508	7.8
NYHA class IV heart failure	196	3.0
Congestive heart failure	704	10.8
Acute coronary syndrome	74	1.1
Ischemic heart disease	789	12.1
Hypertension	1,245	19.1
COPD (on medications)	483	7.4
Chronic renal failure	1,024	15.7
Dialysis	512	7.9
Diabetes mellitus (oral hypoglycemic-dependent insulin)	1,169	17.9
Diabetes mellitus (insulin-dependent)	568	8.7
Elective surgery	939	14.4
Emergency surgery	473	7.3
Alcohol abuse	891	13.7
Autoimmune disease	306	4.7
Organic brain disease	362	5.6
Neuromuscular disease	106	1.6

COPD, chronic obstructive pulmonary disease; NYHA, New York Heart Association. ^a^Presented as mean ± standard deviation.

**Table 2:** Laboratory values and severity of illness characteristics

**Parameter**	**Mean**	**Standard deviation**
APACHE II score	26.1	8.2
Blood assay on day 1		
Creatinine (μmol/l)	219	181
Bilirubin (μmol/l)	41	84
Bicarbonate (mEq/l)	19.4	6.5
Lactate (mmol/l)	4.8	4.4
Platelets (×10^9^/l)	196	139
International Normalized Ratio	1.8	1.4
White blood cell count (×10^6^/l)	16.3	16.1
Heart rate (/minute)	115	29
	**Number**	**Percentage**
Infection characteristics		
Nosocomial	2,594	39.8
Bacteremia/fungemia	2,895	34.6
Culture-positive	4,584	70.4
Primary infection site		
Pulmonary	2,643	40.6
Abdominal/gastrointestinal	1,814	27.8
Urinary	691	10.6
Skin/soft tissue	469	7.2
Central nervous system	54	8.3
Intravascular catheter	224	3.4
Primary bloodstream	379	5.8
Disseminated systemic	135	2.1
Bone and joint	42	0.6
Mediastinal	63	1
Infecting organism		
*Staphylococus aureus*	778	17.0
*Sreptococcus pneumoniae*	350	7.6
Other streptococci	272	5.9
Other Gram-positive cocci	218	4.8
*Escherichia coli*	940	20.5
Other enterobacteriaciae	773	16.9
Nonenterobacteriaciae Gram-negative bacilli	464	10.1
Miscellaneous bacteria	314	6.8
Candida/fungi	474	10.3

APACHE, Acute Physiology and Chronic Health Evaluation.

### Treatment characteristics

The median time to vasopressor initiation was 3 hours (25 to 75% range: 1 to 7.1 hours). The distribution of vasopressor use is presented in Table 3. The most commonly used vasopressor was norepinephrine in about two-thirds of patients, with dopamine being the second most common used in approximately one-half. Use of a given vasopressor was not exclusive of use of others. Dobutamine, an inotropic agent, was used for at least 30 minutes during the first 24 hours after pressor initiation in 12.2% of cases. However, inotropes were never initiated before pressors and an intrope alone was never used (per inclusion criteria). Steroids were used in 32% of patients.

**Table 3:** Treatment and vasopressor use characteristics

**Treatment**	**Number**	**Percentage**
Steroids	1,893	21.8
Activated protein C	292	3.4
Source control required	2,564	39.4
Pressor/inotrope agents used in first 24 hours		
Norepinephrine	4,376	67.2
Dopamine	3,502	53.8
Phenylephrine	1,466	22.5
Dobutamine	793	12.2
Vasopressin	708	10.7
Epinephrine	313	4.8

### Outcomes

**Figure 1:**
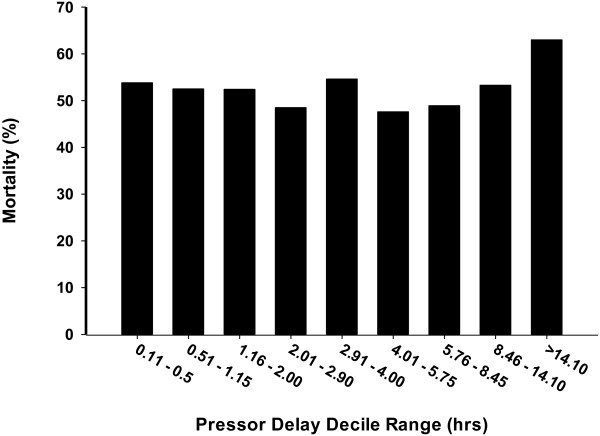
Unadjusted mortality in each pressor delay decile.

The overall unadjusted mortality rate was 53%. Unadjusted mortality among deciles ranged from 47.6% to 63.0% (Figure 1).

### Independent correlates of mortality

The significant independent correlates of mortality from the multivariable analysis are presented in Table 4 in order of descending influence on mortality based on Wald *χ*^2^ values. Among these correlates, the APACHE II score was most significant with an OR of 1.11 per point (95% CI = 1.10 to 1.12). Antimicrobial delay was the next most important variable, each hour of delay was associated with a 7% increase in mortality (OR = 1.07, 95% CI = 1.06 to 1.08) and age was associated with a 2.6% increase in mortality per year of life (OR = 1.03, 95% CI = 1.02 to 1.03). Among categorical variables, liver failure had the strongest association with mortality (OR = 3.46, 95% CI = 2.67 to 4.48). A history of hypertension was found to convey a protective effect (OR = 0.62, 95% CI = 0.52 to 0.73).

**Table 4:** Multivariate correlates of death in septic shock

	**OR**	**95% CI**	***P *** **value**	**Wald *χ***^**2**^
APACHE II score (per point)	1.11	1.10 to 1.12	<0.0001	544.6
Antimicrobial delay (per hour)	1.07	1.06 to 1.08	<0.0001	335.6
Age (per year)	1.03	1.02 to 1.03	<0.0001	127.1
Liver failure	3.46	2.67 to 4.48	<0.0001	88.3
Hypertension	0.62	0.52 to 0.73	<0.0001	32.2
Hematologic malignancy	1.88	1.46 to 2.41	<0.0001	24.1
Metastatic cancer	1.63	1.32 to 2.01	<0.0001	20.4
Vasopressor delay (per hour)	1.02	1.01 to 1.03	0.0099	20.1
Neutropenia	1.78	1.27 to 2.49	0.0008	11.2
AIDS	1.91	1.29 to 2.81	0.0011	10.7

APACHE, Acute Physiology and Chronic Health Evaluation; CI, confidence interval; OR, odds ratio.

**Figure 2:**
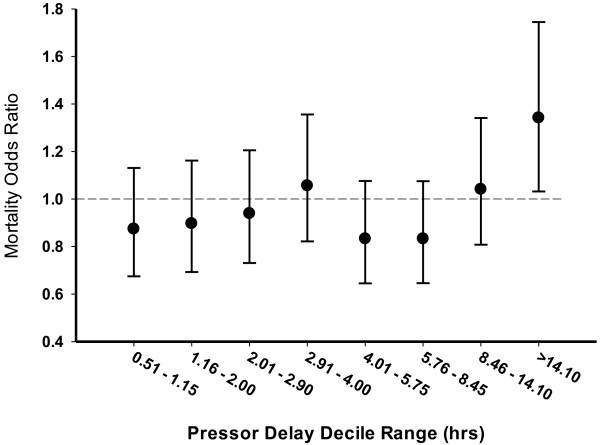
Odds ratio (±95% confidence interval) of mortality for each pressor delay decile (reference decile, 0.11 to 0.5 hours).

After adjusting for independent correlates of mortality (AIDS, hypertension, liver failure, neutropenia, malignancy, metastatic disease, APACHE II score and delay in appropriate antimicrobials), there was a weak association of delay of vasopressors with in-hospital mortality (adjusted OR = 1.02, 95% CI = 1.01 to 1.03, *P* < 0.001). To examine the impact of delays in vasopressor initiation further, deciles of delay were examined in the model. The results are shown in Figure 2. At increasing delays of approximately 0.50 to 1.15 hours, 1.16 to 2.00 hours, 2.01 to 2.90 hours, 2.91 to 4.00 hours, 4.01 to 5.75 hours, 5.76 to 8.45 hours, 8.46 to 14.10 hours and >14.10 hours (reference second decile, 7 to 30 minutes as per the analysis protocol), the adjusted OR of survival was significantly increased only for the final, latest decile (OR = 1.34, 95% CI = 1.03 to 1.76, *P* = 0.048).

### Secondary outcome analysis (organ failure and length of stay)

**Figure 3:**
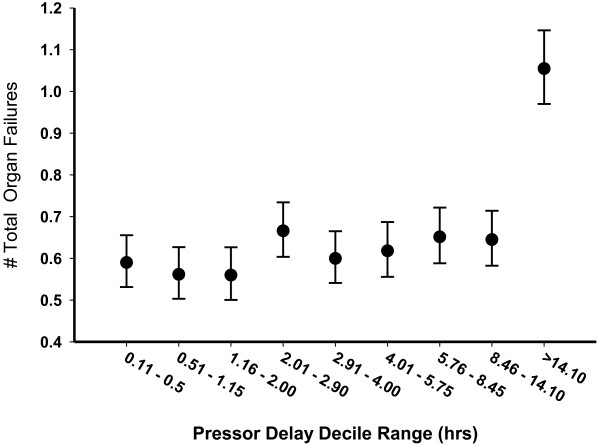
Mean (±95% confidence interval) incremental organ failures (day 2 to day 10 after presentation) with increasing pressor delays.

Secondary outcomes were adjusted for the same independent predictors of mortality as the primary outcome. In both unadjusted and adjusted analyses, a strong trend or actual significance was found between the delay to pressor initiation and the occurrence of organ failures. Adjusted *P* values were as follows: renal, *P* = 0.0182; respiratory, *P* < 0.0001; hematologic, *P* = 0.0788; central nervous system, *P* = 0.0208; coagulation, *P* = 0.0089; metabolic, *P* < 0.0001. Notably, in each case, the last decile (>14.1 hours) accounted for the impact of pressor delay on the occurrence of organ failure. In addition, the total incremental organ failures after the day of presentation (that is, day 2 to day 10) was associated with pressor delay. Again, this relationship was driven by the last decile of delay (Figure 3).

For the survivors, while controlling for significant variables, delay in vasopressor initiation was not predictive of hospital LOS (*P* = 0.19) or ICU LOS (*P* = 0.17). In addition, there was no significant impact on duration of vasopressor/inotropic therapy (*P* = 0.313) and only a trend towards a longer duration of ventilator support (*P* = 0.055) among survivors.

## Discussion

Hypotension is a central feature in the pathophysiology of septic shock. The duration of hypotension before intervention in cardiogenic shock caused by massive myocardial infarction, obstructive shock due to pulmonary embolus and hypovolemic shock due to major trauma/hemorrhage is a key determinant of survival [21-25]. Outcome in these conditions is closely associated with earlier initiation of therapy [21-26]. Similarly, in septic shock, early initiation of fluid resuscitation and rapid administration of appropriate antimicrobials are critical determinants of outcome and central tenets of management [14,27,28]. Based on these factors, we hypothesized that longer duration of hypotension without hemodynamic support using vasopressor infusion may result in a higher mortality rate and an increased incidence of organ failure in septic shock patients.

Our study demonstrates that the interval between diagnosis of septic shock and the administration of vasopressor agents is a significant although modest independent correlate to in-hospital mortality and development of late organ failure. The entire increasing mortality effect with increased delays in vasopressor initiation is related to the increased mortality in the final decile group (>14 hours post hypotension documentation) relative to the reference group. Similarly, increasing probability of incremental aggregate organ failures after the day of shock (that is, day 2 to day 10) is only seen in the highest delay decile groups (>14 hours post hypotension documentation). New-onset renal, respiratory, central nervous system, coagulation and metabolic failures were also individually associated with pressor delays >14 hours. Perhaps because of the modest strength of the correlation between pressor delay and mortality/organ failure, there is no association in the survivor group with ICU or hospital length of stay, ventilator duration or total vasopressor administration time.

Studies have shown that septic shock as defined in part by persistent hypotension is an indicator of a marked increase in morality risk in septic states [29,30]. At least two retrospective human septic shock studies show an increasing mortality with increasing severity and duration of hypotension [31,32]. Varpula and colleagues showed in 111 septic shock patients that the time spent below a MAP of 65 mmHg in the first 48 hours was a strong predictor of mortality [31]. In another retrospective study, Dünser and colleagues similarly measured the area under the curve for MAP and effect on mortality in 274 sepsis patients [32]. This study demonstrated that the time spent with MAP <55 mmHg was associated with increased risk of death. However, a similar correlation did not exist with the duration when MAP was <60 mmHg, <65 mmHg, <70 mmHg and <75 mmHg.

While there has been much study into the comparison of vasopressors/inotropes individually and in combination [33-35], there has been a relative paucity in the literature regarding the timing of their initiation in septic shock. The 2012 Surviving Sepsis Guidelines recommend that vasopressor support be started for fluid-refractory shock as part of the 6-hour bundle based solely on expert opinion [15]. A rat model of endotoxic shock has suggested potential benefit with a higher proportionate splanchnic blood flow, lower lactate levels and less overall fluid support requirement for early compared with delayed norepinephrine administration [36]. A porcine model of fecal peritonitis/shock has demonstrated that delayed resuscitation (inclusive of antibiotics, fluids and pressors) was associated with increased physiologic instability and higher pressor requirements [37]. Conversely, in a small (*n* = 95) retrospective human study, no difference in organ dysfunction or ICU LOS was noted with early (<1.37 hours) versus late (>1.37 hours) administration of vasopressors [16]. These studies have their limitations in that two were animal studies and none utilized survival as an endpoint.

In our study, the timing of initiation of vasopressors following documentation of hypotension is only weakly associated with mortality in septic shock, as indicated by the low Wald *X*^2^ values in Table 4. The Wald *X*^2^ value for delays in antimicrobial initiation, the other remediable treatment parameter in the multivariate analysis, is 16.7 times higher. Note that this does not suggest that duration of hypotension before resuscitation (inclusive of appropriate antimicrobials and fluid resuscitation) is only weakly correlated to outcome. On the contrary, appropriate antimicrobial delays relative to hypotension and early fluid resuscitation are well established to have critical roles in improving outcome of septic shock [14,28]. Only the delay of vasopressors appears to have a limited impact on outcome in this retrospective analysis.

Given the modest strength of the association, the statistical significance of time to vasopressor initiation relates primarily to the extraordinarily large number of cases in this dataset. The only decile group that appears to carry an increased mortality or specific organ failure risk relative to the reference group is the latest group (>14 hours post hypotension documentation). All included deciles to that point appear to carry no significant increased mortality or specific organ failure risk after adjustment for multiple morbid/epidemiologic factors. This finding is entirely congruent with the findings of Subramanian and colleagues, who showed no impact of vasopressor delays up to 12 hours on organ function in a smaller cohort of <100 patients [16].

A history of hypertension conveying a protective effect was an unexpected result on multivariate analysis. It is possible that this finding may be explained by user bias, in that these patients may have activated the healthcare system more frequently to gain a diagnosis of an otherwise silent condition. Hypertension is normally a silent condition, which may suggest that these patients had more routine access to medical care. Alternatively, the study entry criteria (decrease in systolic pressure >40 mmHg) used for many of these patients may be overly sensitive with respect to diagnosing septic shock. The impact of antimicrobial delay on mortality is not surprising because an earlier version of this database demonstrated this same finding [28] and animal studies demonstrate parallel results [38,39].

Overall, the results of this study are congruent with the limited available human data. The study contributes significantly by adding statistical power with a larger sample size while correcting for known confounders (antimicrobial delay, disease severity). There are still significant study limitations. The study did control for delays in antimicrobial administration. However, we were unable to adjust for early fluid administration using this dataset. Although fluid resuscitation is considered a vital part of the initial resuscitation by emergency room physicians and intensivists [15], there are studies suggesting increased mortality associated with over-resuscitation of fluids [40,41]. Other studies conversely suggest increased mortality with under-resuscitation with fluids [14,42]. Significant interactions between the timing of vasopressor initiation and early fluid resuscitation that we are unable to capture in this dataset may exist. This is a significant limitation of this study and future analyses should also attempt to factor in fluid resuscitation.

There are other limitations to this study. This is a retrospective review with its inherent inability to account for all potential confounders. However, there has yet to be a randomized controlled trial of timing of vasopressor initiation in any critical illness. Given the ethical concerns of exposing moribund patients to potential harm, a prospective, randomized human study of timing of vasopressor initiation in septic shock would be challenging. Another limitation is that the use of hypotension as the defining criteria for septic shock in this patient group may be imperfect. MAP is at best a surrogate of inadequate microvascular perfusion in shock. It does not directly capture microcirculatory perfusion and cellular injury that lead to organ dysfunction and death [7,11,13]. Nonetheless, other metabolic markers such as serum lactate and bicarbonate levels as well as severity of illness scores (APACHE II scores) were incorporated into the model to help adjust for variations in shock severity. Despite these limitations of blood pressure monitoring, given its universal access and ease of use it is the most relied upon clinical parameter for guiding therapy and will remain a mainstay in the treatment of septic shock for the foreseeable future.

## Conclusion

From this study, we conclude that markedly delayed initiation of vasopressor medications in patients with septic shock is modestly associated with increased organ failure risk and decreased survival. Substantial delays of vasopressor initiation (>14 hours after hypotension documentation) are required to see these effects. Given the almost universal use of vasopressors in septic shock and the critical need for precise titration, further study of this area is warranted.

## Key messages

•Delays in initiation of vasopressor therapy following the first documentation of hypotension in septic shock are modestly associated with increased specific organ failure and mortality risk.

•This increase in specific organ failure and mortality risk is entirely driven by the decile of patients with the greatest delays of >14 hours.

•Vasopressor initiation delays are not associated with increased time on vasopressors or on mechanical ventilation among survivors.

•Delay of initiation of appropriate antimicrobial, age and APACHE II score are also independent correlates of mortality.

## Abbreviations

APACHE: Acute Physiology and Chronic Health Evaluation; CI: confidence interval; LOS: length of stay; MAP: mean arterial pressure; OR: odds ratio.

## Competing interests

AK received unrestricted funding for the initial development of this database from Lilly, Pfizer, Astellas, Merck and Bayer. Additional support was provided through grants from the Manitoba Health Research Council, the Health Sciences Foundation and the Deacon Foundation. The current analysis/paper was not funded by any sponsor. JEP consulted with Sangart, Artisan, Philips, and Immunetrics. All other authors have no other relevant competing interests.

## Authors’ contributions

AK had full access to all the data in the study and is responsible for the integrity of the database and the accuracy of the data analysis. This specific research concept, the septic shock database and manuscript were developed by AK. AK, DC, AP, GLB and VB were responsible for the methodological design issues and data analysis. AK and VB drafted the manuscript. AK, VB, DC, AP, GLB, SZ and JEP contributed to data interpretation and manuscript revisions. All authors read and approved the final manuscript.

## Supplementary Material

Additional file 1 **Is a list of the additional members of the Cooperative Antimicrobial Therapy of Septic Shock (CATSS) Database Research Group.** List of CATSS Database Research Group Full and Associate Members. Click here for file

Additional file 2 Is a list of participating institutions for study. Click here for file
