# Supplementary material for: Timing of vasopressor initiation and mortality in septic shock: a cohort study
Source: Crit Care. 2014 May 12;18(3):R97. doi: 10.1186/cc13868 (PMC4075345; doi:10.1186/cc13868)
Supplement: Additional file 1 — Is a list of the additional members of the Cooperative Antimicrobial Therapy of Septic Shock (CATSS) Database Research Group. List of CATSS Database Research Group Full and Associate Members. [file cc13868-S1.docx]

**Additional file 1**

**Additional** **Members of the Cooperative Antimicrobial Therapy of Septic Shock (CATSS) Database Research Group**

Yaseen Arabi, MD, King Saud Bin Abdulaziz University for Health Sciences, Riyadh, Saudi Arabia

Phillip Dellinger, MD, Cooper Hospital/University Medical Center, Camden NJ, USA

Sandra Dial, MD, McGill University, Montreal QC, Canada

Peter Dodek, MD, St. Paul’s Hospital, Vancouver, BC, Canada

Paul Ellis, MD, University Health Network, Toronto, ON, Canada

Daniel Feinstein, MD, Moses H. Cone Memorial Hospital, Greensboro NC, USA

Dave Gurka, MD, Rush-Presbyterian-St. Luke’s Medical Center, Chicago IL, USA

Jose Guzman, Cleveland Clinic, Cleveland, OH, USA

Sean Keenan^,^ MD, Royal Columbian Hospital, New Westminster BC, Canada

Andreas Kramer, MD, Foothills Hospital, Calgary AB, Canada

Aseem Kumar, Laurentian University, Sudbury, ON, Canada

Stephen Lapinsky, MD, Mount Sinai Hospital, Toronto ON, Canada

Denny Laporta, MD, Jewish General Hospital, Montreal QC, Canada

Kevin Laupland, MD, Foothills Hospital, Calgary AB, Canada

Bruce Light, MD, Winnipeg Regional Health Authority, Winnipeg MB, Canada

Dennis Maki, MD, University of Wisconsin Hospital and Clinics, Madison WI, USA

John Marshall, MD, St. Michael’s Hospital, Toronto ON, Canada

Greg Martinka, MD, Richmond General Hospital, Richmond BC, Canada

Ziad Memish, MD, Ministry of Health, Riyadh, Saudi Arabia

Yazdan Mirzanejad, MD, Surrey Memorial Hospital, Surrey, BC, Canada

Gourang Patel, PharmD, Rush-Presbyterian-St. Luke’s Medical Center, Chicago IL, USA

Charles Penner, MD, Brandon General Hospital, Brandon MD, Canada

Dan Roberts, MD, Winnipeg Regional Health Authority, Winnipeg MB, Canada

John Ronald, MD, Nanaimo Regional Hospital, Nanaimo BC, Canada

Dave Simon, MD, Rush-Presbyterian-St. Luke’s Medical Center, Chicago IL, USA

Sat Sharma, MD Winnipeg Regional Health Authority, Winnipeg MB, Canada

Nehad Al Shirawi, MD, King Saud Bin Abdulaziz University for Health Sciences, Riyadh, Saudi Arabia

Yoanna Skrobik, MD, Hôpital Maisonneuve Rosemont, Montreal QC, Canada

Gordon Wood, MD, Royal Jubilee Hospital/Victoria General Hospital, Victoria BC, Canada

Kenneth E. Wood, DO, Geisinger Medical Center, Danville PA, USA

**Associate Members of the CATSS Database Research Group**

Muhammed Wali Ahsan, MD, Winnipeg MB, Canada

Mozdeh Bahrainian, MD, Madison WI

Rob Bohmeier, University of Manitoba, Winnipeg MB, Canada

Lindsey Carter, MD, University of Manitoba, Winnipeg MB, Canada

Harris Chou, BSc, of British Columbia, Vancouver BC, Canada

Sofia Delgra, RN, King Saud Bin Abdulaziz University for Health Sciences, Riyadh, Saudi Arabia

Herve Ngawa Djomo, MD, Winnipeg MB, Canada

Collins Egbujuo, MD, Winnipeg MB, Canada

Winnie Fu, University of British Columbia, Vancouver BC, Canada

Catherine Gonzales, RN, King Saud Bin Abdulaziz University for Health Sciences, Riyadh, Saudi Arabia

Harleena Gulati, MD, University of Manitoba, Winnipeg MB, Canada

Oliver Gutierrez, MD, University of Manitoba, Winnipeg MB, Canada

Erica Halmarson, MD, University of Manitoba, Winnipeg MB, Canada

John Hansen, MD, Winnipeg MB, Canada

Ziaul Haque, MD, Montreal QC, Canada

Johanne Harvey, RN, Hôpital Maisonneuve Rosemont, Montreal QC, Canada

Farah Khan, MD, Toronto ON, Canada

Ehsan Koohpayehzadeh, MD, Univesity of Manitoba, Winnipeg, MB, Canada

Laura Kolesar, RN, St. Boniface Hospital, Winnipeg MB, Canada

Laura Kravetsky, MD, University of Manitoba, Winnipeg MB, Canada

Runjun Kumar, University of Toronto, Toronto ON, Canada

Nasreen Merali, MD, Winnipeg MB, Canada

Sheri Muggaberg, University of Manitoba, Winnipeg MB, Canada

Heidi Paulin, University of Toronto, Toronto ON, Canada

Cheryl Peters, RN, MD, University of Manitoba, Winnipeg MB, Canada

Jody Richards, Camosun College, Victoria BC, Canada

Anna Deryl Sagun, MD, Winnipeg MB, Canada

Christa Schorr, RN, Cooper Hospital/University Medical Center, Camden NJ, USA

Honorata Serrano, RN, King Saud Bin Abdulaziz University for Health Sciences, Riyadh, Saudi Arabia

Mustafa Suleman, MD, Concordia Hospital, Winnipeg, MB

Amrinder Singh, MD, Winnipeg MB Canada

Katherine Sullivan, University of Manitoba, Winnipeg MB, Canada

Robert Suppes, MD, University of Manitoba, Winnipeg MB, Canada

Leo Taiberg, MD, Rush Medical College, Chicago IL, USA

Ronny Tchokonte, MD, Wayne State University Medical School, Detroit MI, USA

Omid Ahmadi Torshizi, MD, Montreal QC, Canada

Kym Wiebe, RN, St. Boniface Hospital, Winnipeg MB, Canada
