# Supplementary material for: Timing of vasopressor initiation and mortality in septic shock: a cohort study
Source: Crit Care. 2014 May 12;18(3):R97. doi: 10.1186/cc13868 (PMC4075345; doi:10.1186/cc13868)
Supplement: Additional file 2 — Is a list of participating institutions for study. [file cc13868-S2.docx]

**Additional file 2: Study Institutions**

Health Sciences Centre, Winnipeg MB, Canada

St. Boniface Hospital, Winnipeg MB, Canada

Victoria General Hospital, Winnipeg MB, Canada

Concordia General Hospital, Winnipeg MB, Canada

Grace General Hospital, Winnipeg MB, Canada

Seven Oaks General Hospital, Winnipeg MB, Canada

Brandon General Hospital, Brandon MB, Canada

Richmond General Hospital, Richmond BC, Canada

Surrey Memorial Hospital, Surrey BC, Canada

St. Paul’s Hospital, Vancouver BC, Canada

Royal Columbian Hospital, New Westminster BC, Canada

Nanaimo Regional Hospital, Nanaimo BC, Canada

Royal Jubilee Hospital, Victoria BC, Canada

Victoria General Hospital, Victoria BC, Canada

Foothills Hospital, Calgary AB, Canada

Toronto General Hospital, Toronto ON, Canada

Toronto Western Hospital, Toronto ON, Canada

Mount Sinai Hospital, Toronto ON, Canada

St. Michael’s Hospital, Toronto ON, Canada

Laurentian University, Sudbury, ON, Canada

Hôpital Maisonneuve Rosemont, Montreal QC, Canada

Jewish General Hospital, Montreal QC, Canada

Cooper Hospital/University Medical Center, Camden NJ, USA

Moses H. Cone Memorial Hospital, Greensboro NC, USA

Rush-Presbyterian-St. Luke’s Medical Center, Chicago IL, USA

Cleveland Clinic, Cleveland OH, USA

University of Wisconsin Hospital and Clinics, Madison WI, USA

King Saud Bin Abdulaziz University for Health Sciences, Riyadh, Saudi Arabia
